# Supplementary material for: Effectiveness of Meditation Techniques in Treating Post-Traumatic Stress Disorder: A Systematic Review and Meta-Analysis
Source: Medicina (Kaunas). 2024 Dec 12;60(12):2050. doi: 10.3390/medicina60122050 (PMC11678240; doi:10.3390/medicina60122050)
Supplement: Supplementary file 1 [file medicina-60-02050-s001.zip › Table S1. Study Characteristics and Implementation Statistics for all Studies.pdf]

Appendix A. Part 1. Study Characteristics. Part 2. Implementation Statistics are below.

Mindfulness-Based Other Meditations Study Characteristics

Study ID	*Location*	*Population/Trauma Type*	*Research Design*	*Control Group*	*PTSD Measures*	*Baseline PTSD E*	*Research Quality*	*Mean Age E*	*Sample Total*	*% Males E*	*Study Duration (week)*
**Kim, 2013 MBSB** MB Stretch & Beathe	U of New Mex Health Sciences Center	Nurses with PTSD	RCT	Non-symptom PTSD Base Group	PCL-C, Cortisol, ACTH, DHEAS	43.1	9	47.6	51	9	8, 16
**Heffner, 2016 New York Brief Mindfulness**	U of Rochester and Canandaigua VA Center, NY	Military/ Combat trauma	RCT	TAU	PCL, CAPS, Mindfulness Measures	60 est. Not reported.	8	50	44	89	8
**Heffner, 2016 South Carolina MBPT** Mindfulness Based PTSD	U of Rochester and Canandaigua VA Center, New York	Military/ Combat trauma	RCT	PE	PCL, CAPS	64.9	11	50	17	100	8
**Heffner, 2014 Houston IRV** Inner Resources (Mantra Meditation and Mindfulness)	Michael E. DeBakey VA Medical Center, Houston, TX	Military/ Combat trauma	RCT	PTSD Education	CAPS, PCL	67.2	12	51.5	33	100	8
**Kelly, 2016 TIMBSR** Trauma-Informed MBSR	Quinnipiac Uni versity, Hamden, CT	Female Survivors of Violence	RCT	WLC	PCL-3 BDI-II Beck Depression RSQ	49	10	41.5	45	0	8
**Christopher, 2018 MBRT** MB Resilience Training	U Hillsboro OR School of Graduate Psychology	Police Officers	RCT	NT	Cortisol, Aggression, Organizational stress, burnout, sleep disturbance	Not measured	10	44.73	61	90	12
**King, 2013 MBCT** MB Cognitive Therapy	VA Ann Arbor Michigan	Military/ Combat trauma	CT	TAU	CAPS, PTCI (PTSD-relevant cognitions in self-blame)	74.5	13	60.1	37	100	8
**Heffner, 2014 Richmond MMIP** inter-	Hunter Holmes McGuire VA	Military/ Combat trauma	CT	MM delivered in-person or via	PCL, CAPS	63.2,	10	56.4	52	100	8

person or teleconference	Medical Richmond VA			video conference							
**Shin, 2021 REP-MM**	Hwasun Hospital, Korea	Patients with ileostomy	CT	TAU	PTS	35.1	10	64.2	35	63	12
**Heffner, 2014 Cincinnati MB Cognitive Therapy**	Cincinnati VA Medical Center	Military/ Combat trauma	Single Group	TAU	PCL, CAPS	No data	5	No data	No data	No data	8
**Zalta, 2018 MBSR + Yoga** and CBT	Rush U Medical Center, Chicago	Military/ Combat trauma	Single Group	None	PCL-5, PHQ-9. PTSD	57.13	5	41.4	176	100	3
**Grupe, 2021 MBRT** MB Resilience Training	U of Wisco. Madison Center for Healthy Minds	Police Officers	Single Group	None	PCL, sleep disturbances, anxiety, and burnout	29.9	6	47	30	47	8
**Norman, 2020 MBCBT** Vets and Women Warriors	Weill Institute Neuroscience, U of California SF	Military/ Combat trauma, Sexual Trauma	Single Group	None	PCL-5 (PTSD , PTGI (Post traumatic Grow th Inventory)	Not reported	4	40	10<br>3 Vets<br>7 WW	85<br>0	14 wks Vets,<br>8 wks Women Warriors
**Somohano, 2022 MBRP** Mindfulness-Based Relapse Prevention	Portland VA Medical Center, Portland, VA	Women with PTSD and substance abuse	Single Group	None	PCL-5, PACS (Penn Alcohol Craving Scale)	47.39	5	36.1	23	0	24
**Kirk, 2022 MM + Yoga**, CBT, Web Based	School of Kinesiology and Health Sciences, York Univ. Toronto	Full-time Univer. students	Single Group	None	PCL-5, CAPS, Beck Depression, Anxiety, Pain, 5 Facets of Mindfulness	48.14	7	26.4	22	18	8

Total Sample Size MBO = 636

Inner Resources = Mantra Meditation and Mindfulness

REP-MM = Resilience enhancement with mindfulness meditation

RSQ = The Relationship Structures Questions

Mindfulness-Based Stress Reduction Study Characteristics

Study ID	*Location*	*Population/Trauma Type*	*Research Design*	*Control Group*	*PTSD Measures*	*Baseline PTSD E*	*Research Quality*	*Mean Age E*	*Sample Total*	*% Males E*	*Study Duration (week)*
**Niles, 2012 MBSR**	Veterans Administration (VA) Boston Healthcare System	Military/Combat trauma	RCT	TAU, psycho-education telehealth program	PCL-M, CAPS	52.75	11	52<br>23-66	3	100	8, 14
**Kearney, 2013 MBSR**	VA Puget Sound Health Care System, Seattle	Military/Combat trauma	RCT	TAU	PCL-C PHQ-9 Traumatic Events, HRQOL	59.88	10	52	47	80	12, 24
**Omidi, 2013 MBSR**	Veterans Administration (VA) Boston	Military/Combat trauma	RCT	TAU	DSM-IV (SCID-I criteria interview	No PCL	5	39-59	62	100	8
**Azad, 2014 MBSR**	Behavioral Sciences Research Center, Baqiyatallahh U of Med Sci Mollasadra, Iran	Military/Combat trauma	RCT	TAU, waitlist	Clinical Interview DSM-IV-TR. WHO QOL. and Social Relations	No PCL	6	52.1<br>35-45: 8<br>46-55: 5<br>56-60 1	32	100	4,8
**Polusny, 2015 MBSR**	Minneapolis Veterans Affairs Health Care System	Military/Combat trauma	RCT	Present-centered group therapy	PCL-M, CAPS, WHOQOL-BREF depression: (PHQ-9) mindfulness: (FFMQ)	63.6	12	57.6	116	79	3,6,9,17
**Possemato, 2016 MBSR**	Canandaigua Veterans Affairs Medical Center, NY	Military/ Combat trauma	RCT	TAU	PCL-M, CAPS, PCMHI Meds, PCMHI: Therapy, PHQ-9 (depression). Suicidal Ideation	50.5	11	46.5	62	87.1	8
**Bremner, 2017 MBSR**	Atlanta VA Medical Center	Military/ Combat trauma	RCT	present-centered group therapy (PCGT)	CAPS<br>FFMQ (Five Factor Mindfulness Questionnaire)	56.0	10	34, 35	26	100	8, 24,
**Zhang, 2017 MBSR**	Depart. of Nursing Science, Daqing, China	Breast cancer (BC) patients in China	RCT	TAU	PTGI (PTSD) perceived stress and anxiety	34.54	7	48.67	60	0	12
**Davis, 2018 MBSR**	3 VA Centers Tuscaloosa, AL, Charleston, SC. Atlanta, GA	War Vets/ Military/ Combat trauma	RCT	present-centered group therapy (PCGT)	CAPS	63.1	12	51.5	214	83.3	3,6,9,16
**Kimbrough, 2010 MBSR**	University of Maryland, Baltimore	Child Abuse Survivors	Single Group	None	PCL, BDI-II Depression BSI. GSI, MAAS	46.8	5	44.9	27	11	4,8,24

**Kearney, 2013 MBSR**		Military/ Combat trauma	Single Group	None	PCL-M	52.4	5	51	66	75	8, 24
**Goldsmith, 2014 MBSR**	Rush University Medical Center, Chicago	Trauma Survivors, Mostly Women, Causes not specified.	Single Group	None	PCL-C, PHQ-9, The Child Trauma Q's, Life Events Checklist	34.11	5	44	9	11	8
**Cole, 2015 MBSR**	VA Northern California Health Care System	Military/ Combat trauma, Mild Traumatic Brain Injury	Single Group	None	PCL-C Attention:	63.6	4	45	9	100	12
**Gallegos, 2015 MBSR**	University of Rochester Medical Center	Women with history of interperson trauma	Single Group	None	MPSS-SR MMSE, TLEQ, PSS, DERS, FFMQ, Blood Samples	Not reported	4	44.1	50	0	4, 8, 12

Total Sample Size MBSR = 813
Attention= derived from Cogstate computerized battery.
Blood Samples to determine IL-6 levels (biomarkers of inflammation).
BSI (anxiety)
Depression = Beck Depression Inventory, Second Edition
DERS = Difficulties in Emotion Regulation Scale.
FFMQ = Five Facet Mindfulness Questionnaire.
GSI (General Severity Index)
HRQOL = *Health-related quality of life*. The Short Form-8
MAAS= Mindfulness Attention Awareness Scale
MMSE = Mini Mental State Examination
MPSS-SR= Modified PTSD Symptom Scale Self-Report.
PHQ-9 = Depression
PSS= Perceived Stress Scale.
TLEQ= Traumatic Life Events Questionnaire.
Traumatic Events = Life Events Checklist (LEC; Blake et al., 1995; Gray, Litz, Hsu, & Lombardo, 2004).

Other Meditation Study Characteristics

Study ID	*Location*	*Population/Trauma Type*	*Research Design*	*Control Group*	*PTSD Measures*	*Baseline PTSD E*	*Research Quality*	*Mean Age E*	*Sample Total*	*% Males E*	*Study Duration (week)*
**Bormann, 2008 MR** Mantram Repetition	San Diego and Bedford, Mass VA Med Center	Military/ Combat-trauma	RCT	Usual Care Delayed Treatment	PCL, CAPS	50	8	56	29	100	6
**Bormann, 2013 MR** Mantram Repetition	San Diego VA Medical Center	Military/ Combat-trauma	RCT	TAU	PCL, CAPS	61.39	11	57	146	97	6
**Seppala, 2014 Kriya Yoga** Sudashan Kriya Yoga	Stanford, U of Wisconsin, Madison	Military/ Combat-trauma	RCT	WLC	PCL-M, MASC, Startle	61.4	9	45	21	100	4, 48
**Heffner, 2014 Saginaw AMM** Adopted Mantrum Meditation	Saginaw MI VA	Military/ Combat-trauma	RCT	PCL-M CAPS-M	PCL, CAPS	60.9	12	48.6	22	87	8
**Bormann, 2018 MR** Mantram Repetition	San Diego and Bedford, Mass VA Medical Center	Military/ Combat-trauma	RCT	PCT	PCL, CAPS	59.23	9	48	89	84	8
**Schuurmans, 2020 VRM** Game-Based Meditation	Nijmegen, Netherlands	Trauma youth in residential care	RCT		CRIES-13 Stress, Depress, Anxiety, Aggression	35.8	7	14.46	15	60	4
**Lang, 2019 LKM** Loving Kindness Meditation	Veterans Affairs (VA) San Diego Healthcare System	Military/ Combat-trauma	RCT	Psychoed. about PTSD, relaxation training, and sleep hygiene	CAPS-5, Anxiety, Depression, Emotional distress	35.8	11	49.	28	100	10
**Bayley, 2022 SKY** + Cognitive Processing Therapy	War Related Illness and Injury Study Center, VA Palo Alto	Military/ Combat-trauma	RCT	Cognitive Processing Therapy (CPT)	PCL, BDI, PANAS	59.69	12	57.4	83	83	4, 48
**Kearney, 2021 LKM** Loving-Kindness Meditation	Seattle, Washington VA Medical Center	Military/ Combat-trauma , sexual assault, accident, sudden death	RCT non inferiority trial	Cognitive Process Therapy PTSD.	CAPS-5	34.99	14	56.0	184	76	12, 24
**Church, 2020 Eco Meditation**	National Institute for Integrative Healthcare, Fulton, California	Civilians convenience sample with PTSD	Single Group	None	Two item PCL	55.4	5	55.4	34	12	12
**Heffner, 2014 Loma Linda MR** Mantram Repetition	Loma Linda	Military/ Combat-trauma	Single Group	None	PCL, CAPS	65.7	10	55.4	30	89	8
**Heffner, 2014 Saginaw AMM** Adopted Mantram Repetition	San Diego	Military/ Combat-trauma	Single Group	None	PCL, CAPS	60.9	7	56	20	87	8
**Lang, 2020 LKM** Loving Kindness Meditation	Veterans Affairs (VA) San Diego	Military/ Combat-trauma	Single Group	None	PCL-5, PHQ-9	45.2	4	43.9	23	80	8

**Vasudev, 2020 SKY**	Depart of Psychiatry, Schulich School of Medicine, Western University	Wide range of trauma.	Single Group	None	PCL-5, HAM-D, BDI, HAM-A	44.8	6	51.9	19	15.8	11

Total Sample Size OM = 743

SKY = Kriya Yoga = Sudarshan Kriya Yoga, a Breathing-Based Meditation, CRIES-13 (Verlinden et al., 2014),

TM Study Characteristics

Study ID	*Location*	*Population/Trauma Type*	*Research Design*	*Control Group*	*PTSD Measures*	*Baseline PTSD E*	*Research Quality*	*Mean Age E*	*Sample Total*	*% Males E*	*Study Duration (week)*
**Heffner, 2014 Saginaw TM**	Saginaw MI VA	Military/ Combat- trauma	RCT	AMM, PCT	PCL-M CAPS-M	65.7	7	54.4	65	74	8
**Nidich, 2016 TM**	Oregon State Penitentiary	Civilian-Male Prison Inmates/ Interpersonal violence, psychosocial stress, toxic environment	RCT	TAU	Trauma Check List, Perceived Stress Scale	35.3	10	28.5	181	100	16
**Nidich, 2017 TM**	Coffee Creek Correctional Facility in Wilsonville, OR	Civilian-Female Prison Inmates/ Interpersonal violence, emotional trauma, sexual abuse	RCT	WLC	PCL-C	53.0	9	44.5	20	0	16
**Nidich, 2018 TM**	San Diego VA	Military/ Combat related trauma, disaster exposure, life threatening injury or illness, sexual and other trauma	RCT	PE, HE	PCL-5, CAPS-5	60.5	14	46.4	202	82	4, 6, 8, 10, 13
7 **Bellehsen, 2021 TM**	Northport Veterans Administration Medical Center Long Island, NY	Military/ Combat trauma	RCT	TAU	CAPS 5, PCL 5, BDI,BAI,ISI, Q-LES	53.0	12	52.9	40	80	12
**Leach, 2023 TM**	University of South Australia, Adelaide	Civilian-Abused Women/Domestic Violence	RCT	Facilitated Group Support	PCL-5, DASS-21, AQol-8D	32.7	13	48.2	42	0	8, 16
**Bonamer, 2023 TM**	Sarasota Memorial Health Care System. Moffitt Cancer Center, Tampa General Hospital	Nurses/ Caregiver Stress	RCT	WLC	PCL-5, SFI (17) GAD-7 MBI	28.8	10	42.0	104	0	4, 12

**Brooks, 1985 TM**	VA Denver Colorado, US	Military/ Combat- trauma	CT	PT	DSM III PTSD Criteria	59.8	9	33.3	18	100	12
**Rees, 2013 TM**	Kampala, Uganda	Civilian-Refugees in Congo/ Combat Trauma,	CT	WLC	PCL-C	65.2	9	32.8	42	65	4, 19
**Yoshimura, 2015 Ishinomaki TM**	Sendai, Ishinomaki, Tokyo, Japan	Civilian-Earthquake–Tsunami Victims, Disaster Trauma	CT	NT	Kawai Stress Symptoms Checklist	33.8	6	41.6	239	19	1
**Bandy, 2019 TM**	Johannesburg, South Africa	Civilian-College Students/ sexual and criminal victimization, combat experiences	CT	NT	PCL-C<br>BDI	53.3	8	20.6	68	20.6	2, 4, 15
**Didukh, 2023 TM**	Lubeck, Germany	Civilian-Refugees in Ukraine/ Interpersonal violence	CT	WLC	PCL-C,<br>BDI-II	32.4	8	40.2	80	22.5	4, 8
**Rosenthal, 2011 TM**	Capital Clinical Research Associates, Rockville, MD	Military/ Combat trauma	Single Group	None	PCL-M<br>CAPS-M<br>BDI, QLES-Q	65.2	7	30	5	100	12
**Heffner, 2014 Minneapolis TM**	Minneapolis VA Healthcare System	Military/ Combat trauma	Single Group	None	PCL-M, CAPS-M	64.3	7	61.5	17	94	8
**Rees, 2014 TM**	Kampala, Uganda	Civilian Refugees in Congo/ War Trauma	Single Group	None	PCL-C	68.5	5	33.2	11	73	4
**Herron, 2017 TM**	TM Centers nationally	Military/ Combat trauma	Single Group	None	PCL-M	52.6	4	51	46	85	12
**Kang, 2018 TM**	Minneapolis MN VA	Military/ Combat trauma	Single Group	None	PCL-M, CAPS-M	63.0	6	59	29	79.3	9, 17
**Bonamer, 2019 TM**	Sarasota Memorial Health Care System	Nurses/ Caregiver Stress	Single Group	None	Secondary Traumatic Stress, on ProQOL	28.8		44.1	27	0	16
**Fruchter, 2023 TM**	Ministry of Defense's Rehabilitation Clinic, Israel	Military/ Combat trauma	Single Group	None	CAPS-5, PSQI, ASUQ	45.5	5	50.5	12	100	12, 24

Total Sample # Subjects TM = 1248

Total Sample All Categories: MBO 636 + MBSR 813 + OM 743 + TM 1248 = 3440

Part 2. Implementation Statistics

Mindfulness-Based Other Techniques Implementation Statistics

Study ID	Offered	Learned	% Learned	Completed	% Completed	Dropout	% Dropout	Post Tested	% Post Tested	Regularity
**Kim, 2013 MBSB** MB Stretch & Beathe	11	11	100%	11	100%	0	0%	11	100%	not evaluated
**Heffner, 2016 New York Brief Mindfulness**	44	36	82%	16	36%	28	63%	16	36%	
**Heffner, 2016 South Carolina MBPT** Mindfulness Based PTSD	22	20	91%	11	50%	11	50%	9	41%	not evaluated
**Heffner, 2014 Houston IRV** Inner Resources (Mantra Meditation and Mindfulness)	20	20	100%	17	85%	3	50%	9	41%	
**Kelly, 2016 TIMBSR** Trauma-Informed MBSR	24	19	79%	10	42%	5	21%	19	79%	not evaluated
**Christopher, 2018 MBRT** MB Resilience Training	28	24	86%	24	86%	7	25%	24	86%	Subjects practiced M 10 min/ day, SD 9.52, range 1 to 77 mins.
**King, 2013 MBCT** MB Cognitive Therapy	20	15	75%	15	75%	5	25%	15	75%	102.3 min/wk
**Heffner, 2014 Richmond MMIP** inter-person or teleconference	35	21	60%	21	60%	11	31%	21	60%	In person ave sessions attended 10.3 (85.8%)
**Shin, 2021 REP-MM**	30	27	90%	27	90%	3	10%	27	90%	
CT #2 Mindfulness Telehealth	33	26	79%	31	94%	7	21%	31	94%	Telehealth 8.8 (73.3%)
**Heffner, 2014 Cincinnati** MB Cognitive Therapy	3	3	100%	not reported		not reported		not reported		not evaluated
**Zalta, 2018 MBSR + Yoga** and CBT										
**Grupe, 2021 MBRT** MB Resilience Training	30	30	100%	30	100%	2	7%	28	93%	85%
SG #2 MBRT										
**Norman, 2020 MBCBT** Vets and Women Warriors	23	23	100%	16	70%	7	30%	15	65%	not evaluated
**Somohano, 2022MBRP** Mindfulness-Based Relapse Prevention	83	23	28%	28	34%	60	72%	23	28%	not evaluated
**Kirk, 2022 MM + Yoga**, CBT, Web Based	22	20	91%	18	82%	2	72%	23	28%	
**Sum**	428	318	74%	275	64%	151	35%	271	63%	
**Mean**	28.5	21.2	84%	19.6	72%	10.8	34%	19.4	65%	
**SD**	18.0	7.8	19%	7.2	23%	15.7	24%	7.1	26%	
**Range**	3-190	3-176	28% to 100%	2-176	34% to 100%	0-60	19%	9 to 28	21%	

Mindfulness-Based Stress Reduction Implementation Statistics

Study ID	Offered	Learned	% Learned	Completed	% Completed	Dropout	% Dropout	Post Tested	% Post Tested	Regularity
**Niles, 2012 MBSR**	17	17	100%	13	76%	5	29%	13	76%	>2h/wk during 8 wks
**Kearney, 2013 MBSR**	25	23	92%	21	84%	4	16%	21	84%	not evaluated
**Omidi, 2013 MBSR**	31		not reported	not reported		not reported		not reported		not reported
**Azad, 2014 MBSR**	16	16	100%	14	88%	2	13%	14	88%	not reported
**Polusny, 2015 MBSR**	58	56	97%	45	78%	13	22%	54	93%	not reported
**Possemato, 2016 MBSR**	36	20	56%	16	44%	16	44%	29	81%	not evaluated
**Bremner, 2017 MBSR**	17	9	53%	9	53%	8	47%	9	53%	85%
**Zhang, 2017 MBSR**	30	29	97%	29	97%	2	7%	28	93%	"difficult to persist" 40-45 mins1/ day
**Davis, 2018 MBSR**	107	96	90%	71	66%	35	33%	65	61%	Not evaluated
**Kimbrough, 2010 MBSR**	27	26	96%	23	85%	6	22%	24	78%	Not evaluated
**Kearney, 2013 MBSR**	49	47	96%	69	141%	3	6%	47	96%	Not evaluated
**Goldsmith, 2014 MBSR**	16	10	63%	10	63%	6	38%	9	56%	not evaluated
**Cole, 2015**	16	9	56%	9	56%	8	50%	9	50%	Subjects practiced M 6.4 hrs/wk, SD 4.6, range 2.4 to 17.
**Gallegos, 2015 MBSR**	50	42	84%	24	15%	13	26%	23	46%	50%
**Sum**	**495**	**400**	**81%**	**353**	**71%**	**121**	**24%**	**345**	**70%**	
**Mean**	**35.4**	**30.8**	**83%**	**27.2**	**73%**	**9.3**	**27%**	**26.5**	**75%**	
**SD**	**24.9**	**24.6**	**19%**	**21.4**	**30%**	**8.9**	**15%**	**18.2**	**18%**	
**Range**	**16-107**	**9--96**	**53%-100%**	**Sep-71**	**13% to 100%**	**2--35**	**82% to 0%**	**9---65**	**46% to 100%**	

Other Meditation Implementation Statistics

Study ID	Offered	Learned	% Learned	Completed	% Completed	Dropout	% Dropout	Post Tested	% Post Tested	Regularity
**Bormann, 2008 MR** Mantram Repetition	33	29	88%	29	88%	4	12%	29	88%	not reported
**Bormann, 2013 MR** Mantram Repetition	71	69	97%	66	93%	5	7%	29	41%	7.6 sessions/day
**Seppala, 2014 Kriya Yoga** Sudashan Kriya Yoga	11	11	100%	10	91%	2	18%	9	82%	
**Heffner, 2014 Saginaw AMM** Adopted Mantrum Meditation	25	25	100%	24	96%	3	12%	22	88%	7.6 (95%) attended the 8 training sessions

**Bormann, 2018 MR** Mantram Repetition	89	69	78%	69	78%	24	27%	65	73%	completers 10 sessions/day, 6 days/week
**Schuurmans, 2020 VRM** Game-Based Meditation	15	12	80%	11	73%	4	27%	11	73%	
**Lang, 2019 LKM** Loving Kindness Meditation	37	31	84%	19	51%	18	49%	26	70%	
**Bayley, 2022 SKY** + Cognitive Processing Therapy	41	30	73%	30	73%	11	27%	26	63%	
**Kearney, 2021 LKM** Loving-Kindness Meditation	91	61	67%	61	67%	30	33%	61	67%	
**Church, 2020 Eco Meditation**	340	299	88%	340	100%	200	59%	140	41%	posttested at 6 mos.
**Heffner, 2014 Loma Linda MR** Mantram Repetition	37	37	100%	30	81%	7	19%	27	73%	6 of 8 lessons completed
**Lang, 2020 LKM** Loving Kindness Meditation	37	31	84%	19	51%	18	49%	26	70%	7 did 1-3 sessions, 5 did 4-5
**Vasudev, 2020 SKY**	33	21	64%	21	64%	14	42%	19	58%	
**Sum**	**860**	**725**	**88%**	**729**	**85%**	**340**	**40%**	**490**	**57%**	
**Mean**	**66.2**	**55.8**	**85%**	**56.1**	**77%**	**26.2**	**29%**	**37.7**	**68%**	
**SD**	**86.1**	**75.6**	**12%**	**87.6**	**16%**	**53.0**	**16%**	**34.8**	**15%**	
**Range**	**11-340**	**11-299**	**64%-100%**	**10-340**	**51%-100%**	**2-200**	**7%-59%**	**9-140**	**41%-88%**	

Transcendental Meditation Implementation Statistics

Study ID	Offered	Learned	% Learned	Completed	% Completed	Dropout	% Dropout	Post Tested	% Post Tested	Regularity
**Heffner, 2014 Saginaw TM**	27	21	78%	19	70%	6	22%	19	70%	Monitored but not reported
**Nidich, 2016 TM**	90	90	100%	79	88%	12	13%	73	81%	
**Nidich, 2017 TM**	11	10	91%	10	91%	1	9%	10	91%	81%
**Nidich, 2018 TM**	68	62	91%	51	75%	6	9%	53	78%	76%
**Bellehsen, 2021 TM**	20	20	100%	20	100%	1	5%	19	95%	85%
**Leach, 2023 TM**	21	21	100%	17	81%	4	19%	17	81%	53.2%
**Bonamer, 2023 TM**	53	47	89%	47	89%	0	0%	40	75%	97.5%
**Brooks, 1985 TM**	14	13	93%	13	93%	3	21%	10	71%	Not Evaluated
**Rees, 2013 TM**	21	21	100%	21	100%	0	0%	21	100%	50%
**Yoshimura, 2015 Ishinomaki TM**	497	497	100%	497	100%	0	0%	497	100%	Not evaluated
**Bandy, 2019 TM**	34	34	100%	34	100%	2	6%	32	94%	73%
**Didukh, 2023 TM**	50	40	80%	40	80%	10	20%	40	80%	85%
**Rosenthal, 2011 TM**	7	6	86%	6	86%	2	29%	5	71%	90%
**Heffner, 2014 Minneapolis TM**	21	21	100%	19	90%	2	10%	17	81%	not evaluated

**Rees, 2014 TM**	11	11	100%	11	100%	0	0%	11	100%	not formally evaluated, but "high"
**Herron, 2017 TM**	46	46	100%	46	15%	15	33%	46	100%	100%
**Kang, 2018 TM**	29	29	100%	23	79%	5	17%	23	79%	Not evaluated
**Bonamer, 2019 TM**	27	27	100%	24	89%	3	11%	20	74%	98%
**Fruchter, 2023 TM**	66	12	18%	12	18%	54	82%	23	35%	No dropouts due to irregular practice
**Sum**	**1113**	**1028**	**92%**	**989**	**89%**	**126**	**11%**	**976**	**88%**	**81%**
**Mean**	**58.6**	**54.1**	**91%**	**52.05**	**81%**	**6.63**	**16%**	**51.37**	**82%**	
**SD**	**108.5**	**109.2**	**19%**	**109.3**	**24%**	**12.2**	**19%**	**109.3**	**16%**	
**Range**	**7-497**	**6-497**	**18%-100%**	**6-497**	**18%-100%**	**0-54**	**0%-82%**	**5-497**	**35% -100%**	
