# Supplementary material for: Effectiveness of Meditation Techniques in Treating Post-Traumatic Stress Disorder: A Systematic Review and Meta-Analysis
Source: Medicina (Kaunas). 2024 Dec 12;60(12):2050. doi: 10.3390/medicina60122050 (PMC11678240; doi:10.3390/medicina60122050)
Supplement: Supplementary file 1 [file medicina-60-02050-s001.zip › Table S2. Meditation Modified CLEAR Assessment .pdf]

Appendix B. Meditation Modified CLEAR assessment worksheet
The items highlighted in yellow are the 7 items used in CLEAR2, which is research quality items that are independent of the control group.

Yes is scored 1, No or Unclear is scored 0.

Criterion	Yes	No	Unclear

1 Did the study have a control group?

2 Was the generation of allocation sequences (group randomization assignment procedure) adequate? Randomization yields groups that are balanced with regard to variables that have an impact the outcome under study (prognostic variables). Prognostic means serving to predict the likely outcome of a disease or ailment; relating to a medical prognosis.

3 Did the study use objective measurement tools for assessment of the outcome? (For example, for PTSD, use of the CAPS would give a yes, use of PCL would give a 'no' because the PCL is a subjective self-report measure.)

4 Did the study use a validated measure(s) of the outcome? (For example, for PTSD, use of either CAPS or PCL would give a yes because both are validated measures of PTSD.) The PCL-5 is a 20-item self-report measure that assesses the 20 DSM-5 symptoms of PTSD. Correlations between total scores on the CAPS and PCL have ranged from . 30 to . 93 (Adkins et al., 2008; Blanchard et al., 1996; Bollinger et al., 2008; Forbes et al., 2001; Keen et al., 2008).

5 Did the study use the same or equivalent measures in pretest and posttest phases? No indicates *Instrumentation threat:* Use of non-equivalent measures at pretest and posttest

6 Did the study measure pretest and posttest in the same location? Did it measure experimental and control groups in the same location? No to either of these questions would give a No for this item.

7 Was participant adherence or compliance assessed quantitatively?

8 Were withdrawals and lost-to-follow-up the same in each randomized or non-randomized

group?

9 Was the follow-up schedule the same in each group? (parallel design)

10 Were care providers for the experimental (meditation) group experienced and skilled? (For example, did the study use a qualified teacher of the meditation technique?)

11 Were care providers for the control group experienced or skilled? (For example, did the study use a qualified teacher of the control or comparison condition?)

12 Did the study have systematic follow-up and checking of correct practice of the meditation technique?

13 Did the study have systematic follow-up and checking of correct practice of the control or comparison condition?

14 Were the treatment and control groups comparable at entry, i.e., were there any significant differences at baseline? (If there were differences, but they covaried for them in reporting the results, we are giving a 'Yes', indicating that the pretest measures were equivalent. If there were no differences in pretest measured = Yes. It there were differences but they were covaried for = Yes. If there were differences but not covaried for = No, 0.)

15 Were the main outcomes analyzed according to the intention-to-treat principle? Applying the intention-to-treat principles yields an unbiased estimate of the efficacy of the intervention on the primary study outcome at the level of adherence observed in the trial.

Total= Total scores (number of Yes's)
